# Supplementary material for: Diffusion-MRI-based regional cortical microstructure at birth for predicting neurodevelopmental outcomes of 2-year-olds
Source: eLife. 2020 Dec 22;9:e58116. doi: 10.7554/eLife.58116 (PMC7755384; doi:10.7554/eLife.58116)
Supplement: Supplementary file 1. — aC for C-section and V for vaginal birth; bB for breast-feeding and F for formula; wks: postmenstrual weeks. [file elife-58116-supp1.docx]

**Supplementary file 1:** Demographics of participating subjects who went through MRI scans at birth and was assessed with Bayley tests at their 2 years of age. ^a^C for C-section and V for vaginal birth; ^b^ B for breast-feeding and F for formula; wks: postmenstrual weeks.


|  | | **Age range (wks)** | **Age mean (wks)** | **Age**  **>37wks**  **n(%)** |  | **Weight range (kgs)** | **Weight mean (kgs)** | **Male n(%)** | **White n(%)** | **Mode of delivery ^a^** | **Feeding practice ^b^** | **Antibiotic exposure during pregnancy** |
| --- | --- | --- | --- | --- | --- | --- | --- | --- | --- | --- | --- | --- |
| Cohort with MRI scan  (n=87) | At birth | 26.0-41.4 | 33.6 | 23(26) |  | 0.8-4.1 | 2.1 | 58 (67) | 61(70) | C:33;  V:54 | B: 87;  F: 0 | Yes |
|  | At  scan | 31.9-41.7 | 37.0 | 41(47) |  | 1.4-4.1 | 2.5 |  |  |  |  |  |
| Cohort with Bayley exam  (n=46) | At  birth | 25.0-41.4 | 32.4 | 8(17) |  | 0.8-3.9 | 1.8 | 33 (72) | 34(74) | C: 17;  V: 29 | B: 46;  F: 0 | Yes |
|  | At  scan | 31.9-41.7 | 36.7 | 17(37) |  | 1.4-3.9 | 2.4 |  |  |  |  |  |
