# Supplementary material for: Diffusion-MRI-based regional cortical microstructure at birth for predicting neurodevelopmental outcomes of 2-year-olds
Source: eLife. 2020 Dec 22;9:e58116. doi: 10.7554/eLife.58116 (PMC7755384; doi:10.7554/eLife.58116)
Supplement: Supplementary file 2. — Bayley-III neurodevelopmental assessments of the 46 infants as well as r and p-values of the correlation between one of the composite scores and a specific age (birth age, scan age, or Bayley-III assessment age) in both preterm and term born infant groups. [file elife-58116-supp2.docx]

**Supplementary file 2:** Mean and standard deviation of the composite scores from the 2-year Bayley-III neurodevelopmental assessments of the 46 infants as well as r and p values of the correlation between one of the composite scores and a specific age (birth age, scan age or Bayley-III assessment age) in both preterm and term born infant groups.

| **Bayley scale** | **n (>85)** | **Mean (SD)** | **Correlation with age** | | | | | |
| --- | --- | --- | --- | --- | --- | --- | --- | --- |
|  |  |  | **Birth age** | | **Scan age** | | **Bayley assessment age** | |
|  |  |  | preterm | term | preterm | term | preterm | term |
|  |  |  | *r* (p) value | *r* (p) value | *r* (p) value | *r* (p) value | *r* (p) value | *r* (p) value |
| Cognitive | 46 (22) | 87.4 (8.5) | -0.16(0.3) | 0.06(0.9) | 0.23(0.2) | 0.15(0.7) | 0.04(0.8) | -0.07(0.9) |
| Language | 46 (24) | 85.7 (10.1) | -0.07(0.7) | 0.40(0.3) | 0.28(0.1) | 0.52(0.2) | -0.03(0.8) | -0.15(0.7) |
| Motor | 46 (37) | 91.2 (7.1) | -0.04(0.8) | -0.12(0.8) | 0.25(0.1) | -0.09(0.8) | -0.02(0.9) | 0.02(0.9) |
