# Supplementary material for: Diffusion-MRI-based regional cortical microstructure at birth for predicting neurodevelopmental outcomes of 2-year-olds
Source: eLife. 2020 Dec 22;9:e58116. doi: 10.7554/eLife.58116 (PMC7755384; doi:10.7554/eLife.58116)
Supplement: Supplementary file 3. — The top 10 highest weights in each model are marked in bold. [file elife-58116-supp3.docx]

**Supplementary file 3:** Normalized feature contribution weights of cortical FA measures from each gyrus in cognitive and language prediction models. The top 10 highest weights in each model are marked in bold.

| Lobes/cortices | Gyri |  | Cognitive prediction | | Language prediction | |
| --- | --- | --- | --- | --- | --- | --- |
|  |  | Gyrus abbreviation | Left Hemisphere | Right Hemisphere | Left Hemisphere | Right Hemisphere |
| Frontal lobe | Superior frontal gyrus | SFG | 0.014 | 0.011 | 0.008 | 0.001 |
|  | Middle frontal gyrus | MFG | 0.001 | 0.023 | 0.027 | 0.019 |
|  | Inferior frontal gyrus | IFG | 0.003 | 0.006 | **0.044** | 0.026 |
|  | Medial fronto-orbital gyrus | MFOG | 0.016 | **0.043** | 0.015 | **0.041** |
|  | Lateral fronto-orbital gyrus | LFOG | 0.005 | **0.050** | 0.014 | **0.032** |
|  | Gyrus rectus | REG | **0.055** | **0.036** | **0.077** | 0.020 |
|  | Precentral gyrus | PrCG | 0.023 | 0.005 | 0.007 | 0.003 |
| Parietal lobe | Postcentral gyrus | PoCG | **0.050** | 0.033 | **0.052** | 0.003 |
|  | Superior parietal lobule | SPL | 0.024 | 0.021 | 0.018 | 0.021 |
|  | Precuneus | PrCu | 0.003 | **0.042** | 0.016 | 0.007 |
|  | Supramarginal gyrus | SMG | 0.009 | 0.005 | 0.010 | 0.021 |
|  | Angular gyrus | ANG | 0.017 | 0.020 | 0.004 | **0.033** |
| Temporal lobe | Superior temporal gyrus | STG | 0.008 | 0.018 | 0.010 | 0.028 |
|  | Middle temporal gyrus | MTG | 0.011 | 0.006 | 0.008 | 0.020 |
|  | Inferior temporal gyrus | ITG | 0.015 | 0.014 | 0.017 | 0.004 |
|  | Fusiform gyrus | FuG | **0.049** | 0.018 | 0.024 | 0.006 |
| Occipital lobe | Superior occipital gyrus | SOG | 0.022 | 0.003 | 0.009 | 0.015 |
|  | Middle occipital gyrus | MOG | 0.007 | 0.026 | 0.001 | 0.027 |
|  | Inferior occipital gyrus | IOG | 0.008 | 0.011 | 0.003 | 0.029 |
|  | Cuneus | Cu | 0.011 | 0.000 | 0.010 | 0.003 |
|  | Lyngual gyrus | LG | 0.009 | 0.013 | 0.019 | 0.014 |
| Limbic lobe | Cingular gyrus | CingG | 0.031 | 0.010 | **0.029** | 0.004 |
|  | Parahippocampal gyrus | PHG | 0.030 | **0.051** | 0.003 | 0.027 |
|  | Entorhinal cortex | ENT | **0.042** | **0.040** | **0.042** | **0.048** |
|  | Hippocampus | Hippo | 0.008 | 0.000 | 0.014 | 0.027 |
| Insular | Insular cortex | Ins | 0.021 | 0.006 | **0.039** | 0.002 |
