# Supplementary material for: Diffusion-MRI-based regional cortical microstructure at birth for predicting neurodevelopmental outcomes of 2-year-olds
Source: eLife. 2020 Dec 22;9:e58116. doi: 10.7554/eLife.58116 (PMC7755384; doi:10.7554/eLife.58116)
Supplement: Transparent reporting form [file elife-58116-transrepform.docx]

***eLife’s* transparent reporting form**

We encourage authors to provide detailed information *within their submission* to facilitate the interpretation and replication of experiments. Authors can upload supporting documentation to indicate the use of appropriate reporting guidelines for health-related research (see [EQUATOR Network](http://www.equator-network.org/%20)), life science research (see the [BioSharing Information Resource](https://biosharing.org/)), or the [ARRIVE guidelines](http://www.plosbiology.org/article/info:doi/10.1371/journal.pbio.1000412) for reporting work involving animal research. Where applicable, authors should refer to any relevant reporting standards documents in this form.

If you have any questions, please consult our Journal Policies and/or contact us: [editorial@elifesciences.org](mailto:editorial@elifesciences.org).

**Sample-size estimation**

- You should state whether an appropriate sample size was computed when the study was being designed
- You should state the statistical method of sample size computation and any required assumptions
- If no explicit power analysis was used, you should describe how you decided what sample (replicate) size (number) to use

This information can be found in the *Materials and Methods* section and *Supplementary file 1*. Specifically, 107 neonates were recruited from the Parkland Hospital and scanned at Children’s Medical Center at Dallas. Evaluable MRI was obtained from 87 neonates (58 M/ 29 F; MRI scan age: 31.9 to 41.7 postmenstrual week (PMW)). Out of 87 neonates with evaluable MRI scanned around birth, 46 infants (32M/14F, MRI scan age: 31.9 to 41.7PMW) underwent neurodevelopmental assessment at their 2 years of age (20-29months corrected for prematurity, 23.5±2.3months). For this study, the more sample size the better. So all evaluable datasets from 46 infants, specifically evaluable MRI around birth and neurodevelopmental assessment at 2 years of age, were included in prediction procedures.

Please outline where this information can be found within the submission (e.g., sections or figure legends), or explain why this information doesn’t apply to your submission:

**Replicates**

- You should report how often each experiment was performed
- You should include a definition of biological versus technical replication
- The data obtained should be provided and sufficient information should be provided to indicate the number of independent biological and/or technical replicates
- If you encountered any outliers, you should describe how these were handled
- Criteria for exclusion/inclusion of data should be clearly stated
- High-throughput sequence data should be uploaded before submission, with a private link for reviewers provided (these are available from both GEO and ArrayExpress)

Details about replicates include 1) Criteria for exclusion/inclusion of infant recruitment provided in *Materials and Methods: Participants* section; 2) Quality control and quality assurance of MRI provided in the *Materials and Methods*; 3) Diffusion MRI dataset exclusion criteria provided in the *Materials and Methods: Measurement of cortical microstructure with brain MRI at birth*; 4) Assessing robustness of prediction across different parcellation schemes and after age adjustment provided in *Materials and Methods: Assessment of robustness of prediction*; and 5) Assessing reproducibility of prediction results provided in the *Materials and Methods: Bootstrap analysis for assessing reproducibility of top 10 cortical regions identified by LOOCV analysis* section as well as reproducible and distinguishable regional contribution to predicting cognitive or language outcome in Figure 3-figure supplement 1.

Please outline where this information can be found within the submission (e.g., sections or figure legends), or explain why this information doesn’t apply to your submission:

**Statistical reporting**

- Statistical analysis methods should be described and justified
- Raw data should be presented in figures whenever informative to do so (typically when N per group is less than 10)
- For each experiment, you should identify the statistical tests used, exact values of N, definitions of center, methods of multiple test correction, and dispersion and precision measures (e.g., mean, median, SD, SEM, confidence intervals; and, for the major substantive results, a measure of effect size (e.g., Pearson's r, Cohen's d)
- Report exact p-values wherever possible alongside the summary statistics and 95% confidence intervals. These should be reported for all key questions and not only when the p-value is less than 0.05.

Please outline where this information can be found within the submission (e.g., sections or figure legends), or explain why this information doesn’t apply to your submission:

Statistical analysis methods are described throughout the *Results* and more details can be found in the *Materials and Methods: Prediction of neurodevelopmental outcome with cortical FA as features*, *Bootstrap analysis for assessing reproducibility of top 10 cortical regions identified by LOOCV analysis,* and *Permutation tests to assess distinguishable regional contribution to predicting cognitive or language outcomes* sections*.*

(For large datasets, or papers with a very large number of statistical tests, you may upload a single table file with tests, Ns, etc., with reference to sections in the manuscript.)

**Group allocation**

- Indicate how samples were allocated into experimental groups (in the case of clinical studies, please specify allocation to treatment method); if randomization was used, please also state if restricted randomization was applied
- Indicate if masking was used during group allocation, data collection and/or data analysis

We have only one group of subjects in predicting continuous neurodevelopmental outcomes analysis. The only group categorization (group with normal scores vs. group with low scores) was based on widely used subjects’ Bayley-III cut-off score for developmental delay in the classification analysis. Random sampling of data was conducted during permutation tests and bootstrap analysis. More details can be found in the corresponding in *Materials and Methods* and *Results* sections.

Please outline where this information can be found within the submission (e.g., sections or figure legends), or explain why this information doesn’t apply to your submission:

**Additional data files (“source data”)**

- We encourage you to upload relevant additional data files, such as numerical data that are represented as a graph in a figure, or as a summary table
- Where provided, these should be in the most useful format, and they can be uploaded as “Source data” files linked to a main figure or table
- Include model definition files including the full list of parameters used
- Include code used for data analysis (e.g., R, MatLab)
- Avoid stating that data files are “available upon request”

Please indicate the figures or tables for which source data files have been provided:

Normalized feature contribution weights of each cortical gyrus shown in Figure 2 and Figure 3 can be found in the Supplementary file 3. Cortical fractional anisotropy (FA) distributions across parcellated cortical gyri in the left and right hemisphere from all 46 infants who went through a follow-up visit at their 2 years of age can be found in Figure 1-figure supplement 1 and Figure 1-figure supplement 2. Neurodevelopmental scores from these 46 subjects at their 2-year-old can be found in Supplementary file 2 and Figure 1-figure supplement 3. MRI datasets including diffusion MRI of 23 term-born neonates are publicly available and can be freely downloaded from brainmrimap.org (a public website maintained by Huang lab). Other MRI datasets and complete behavioral tests will be made available in the same website brainmrimap.org. Investigators can also contact hao.huang@pennmedicine.upenn.edu to request those datasets before they are made public. Scripts used for prediction are available from first author’s github repository (https://github.com/MHouyang/Prediction-of-neurodevelopmental-outcome).
